# Editorial Comment: Improvement of fertility parameters with Tribulus Terrestris and Anacyclus Pyrethrum treatment in male rats

**DOI:** 10.1590/S1677-5538.IBJU.2018.0843.1

**Published:** 2019-01-29

**Authors:** Diogo Benchimol de Souza, Gabriela Faria Buys-Gonçalves

**Affiliations:** 1 Universidade Estadual do Rio de Janeiro Rio de Janeiro RJ Brasil Unidade de Pesquisa Urogenital Universidade Estadual do Rio de Janeiro - Uerj, Rio de Janeiro, RJ, Brasil

Herbal medicine is as old as the history of mankind, and is still a topic of interest in current days. The article of Haghmorad et al. (1) reports promising results with two herbal extracts for improving fertility parameters. Both herbs showed positive results when used individually, but (what was more interesting) a synergic effect seems to occur when used together. The extract of *Tribulus terrestris* were more prominent in raising LH and Testosterone levels (which was already reported (2,3)) while *Anacyclus pyrethrum* showed more impressive results in raising FSH and improving sperm parameters. Thus, the combined use may improve fertility parameters by two different endocrine ways. One limitation not raised by the authors is that the extracts improved fertility parameters in control animals, in which a normal testicle, hypothalamus-pituitary-gonadal axis and fertility parameters are assumed. Future studies investigating if these herbal extracts can also improve fertility parameters in infertile/subfertile models are warranted.

The mechanisms of action of these phytotherapics are poorly understood, especially for the less-studied Anacyclus pyrethrum. This herb has been proposed for different conditions (from local anesthetic to anticancer (4,5)), although no clinical study was conducted focusing on male reproductive or endocrine systems. It seems that most phytotherapeutic study focuses only on the final specific effects, putting aside the search for knowledge on the basic mechanisms of the extracts.

Since the ancient Greece Hippocrates advocated the principle of *primum non nocere* which should be always applied when proposing any therapy, including herbal therapies. When studying any treatment for a specific condition, is important to have a more global perspective, evaluating potential side-effects of the proposed medication. Specifically, for *Tribulus terrestris*, our group recently showed this herb leads to arterial blood pressure increase and renal morphology alteration with glomerular loss (6). This kind of study may add information for the physician, helping evaluating the pros and cons of each prescription for each patient.
